# LINC01123 facilitates proliferation, invasion and chemoresistance of colon cancer cells

**DOI:** 10.1042/BSR20194062

**Published:** 2020-08-07

**Authors:** Shicai Ye, Bilan Sun, Weiyun Wu, Caiyuan Yu, Ting Tian, Zhongxiong Lian, Qianyi Liang, Yu Zhou

**Affiliations:** 1 Department of Gastroenterology, The Affiliated Hospital of Guangdong Medical University, Zhanjiang 524001, Guangdong, China; 2 Department of Cancer Center, The Affiliated Hospital of Guangdong Medical University, Zhanjiang 524001, Guangdong, China

**Keywords:** colon cancer, LINC01123, miR-34c-5p, VEGFA

## Abstract

Colon cancer is one of the major causes of cancer-related deaths worldwide. Long non-coding RNA (lncRNA) LINC01123 has been suggested to act as an oncogene in non-small cell lung cancer and a prognostic signature in head and neck squamous cell carcinoma. However, its role in colon cancer remains obscure. From TCGA database, LINC01123 was observed to be up-regulated in colon adenocarcinoma (COAD). Subsequently, the up-regulated LINC01123 was also detected in colon cancer cells. Functionally, LINC01123 could enhance cell proliferation, migration, invasion and angiogenesis. Moreover, the chemoresistance of colon cancer cells was verified to be promoted by LINC01123. Afterward, LINC01123 was found to bind with Ago2 and miR-34c-5p. Besides, miR-34c-5p was confirmed to inhibit the cellular process and chemoresistance of colon cancer cells. Then, VEGFA was disclosed to coexist with LINC01123 and miR-34c-5p in RNA-induced silencing complex. And TCGA database suggested that its expression was correlated with different stages of COAD. Moreover, it was uncovered that VEGFA could bind with miR-34c-5p and its expression positively correlated with LINC01123 expression. Finally, LINC01123 was proofed to regulate colon cancer progression and cells chemoresistance via VEGFA. In conclusion, LINC01123/miR-34c-5p/VEGFA axis promotes colon cancer malignancy and cells chemoresistance.

## Introduction

Featured with late tumor occurrence, rapid development, and metachronous metastasis, colon cancer is the third leading cause of cancer-related deaths worldwide [1]. It has been predicted that the cases of colon cancer would increase to 2.2 million and the death number would rise to 1.1 million [2]. Radio- and chemotherapy are the first choice for treatment of the resectable and advanced colon cancer [3,4]. The transformation from normal colonic mucosa to colon cancer involves a progression of accumulating genetic changes [5]. 5-Fluorouracil (5-FU) is a common chemotherapeutic drug using for colon cancer treatment [6]. Hence, exploring the mechanisms underlying genetic changes and radio- or chemoresistance of colon cells is of substantial importance.

Long non-coding RNAs (lncRNAs) are functional RNA molecules, characterized by the length of over 200 nucleotides [7]. LncRNAs are incapable of encoding proteins but could take part in the regulation of gene expression at various levels [8]. At the transcriptional level, lncRNAs could epigenetically regulate the expression of genes by recruiting EZH2. At the post-transcriptional level, lncRNAs could interact with RNA binding proteins to mediate the stability of mRNAs. Besides, lncRNAs could also antagonize the availability of microRNA (miRNA) to free the messenger RNA (mRNA) from the regulation of miRNA [9]. A group of lncRNAs have been discovered to participate in the regulation of colon cancer progression, such as B3GALT5-AS1 [10], SNHG15 [11], HOXB-AS3 [12] and CCAT2 [13]. LINC01123 has been identified as a prognostic biomarker for head and neck squamous cell carcinoma [14]. Moreover, LINC01123 has been revealed to be transcriptionally activated by c-Myc and could act as a molecular sponge of miR-199a-5p to enhance cell proliferation in non-small cell lung cancer [15]. Nevertheless, limited papers have discussed the function of LINC01123 in colon cancer. Therefore, we proposed a hypothesis that LINC01123 might serve as a competing endogenous RNA (ceRNA) in colon cancer.

The primary purpose of this literature was to probe for the functions that LINC01123 exerts in colon cancer progression. Besides, we also investigated whether LINC01123 influences the resistance of colon cancer cells to 5-FU.

## Materials and methods

### Cell culture and treatment

Four human colon cancer cell lines (HCT116, SW620, SW480, LoVo) and human-derived colonic epithelial NCM460 cell line, from the ATCC (Manassas, VA), were allowed to propagate in RPMI medium (Invitrogen, Carlsbad, CA) at 37°C with 5% CO_2_. Medium supplements contained 1% Pen/Strep mixture and 10% FBS (Gibco, Grand Island, NY). LoVo and SW620 cell samples were treated with 5-FU (Sigma–Aldrich, St. Louis, MI) at the increasing concentrations of 0, 20, 40, 60, 80 and 100 μM for 72 h.

### Quantitative real-time PCR

Total RNAs were prepared from the colon cancer cell samples by use of TRIzol reagent (Invitrogen) for reverse transcription. The abundance of transcripts was monitored by qPCR employing SYBR Green PCR Master Mix (Takara, Toyobo, Japan), with U6 or GAPDH as internal control. RNA expression was calculated by 2^−ΔΔ*C*_t_^ method. All primer sequences are listed in Supplementary Table S1.

### Transfection

The duplex LINC01123-specific shRNAs and nonspecific shRNAs as negative control (NC), were procured from GenePharma (Shanghai, China) and transfected into cell samples for 48 h employing Lipofectamine 2000 (Invitrogen). The miR-34c-5p mimics/inhibitor and NC mimics/inhibitor, as well as pcDNA3.1/LINC01123, pcDNA3.1/VEGFA and NC pcDNA3.1 vector were also acquired from GenePharma. Sequences for all plasmids were listed in Supplementary Table S2.

### CCK-8 assay

After transfection, cell samples of colon cancer were exposed to 10 μM of 5-FU for 72 h, then planted into 96-well plates at 1 × 10^4^ cells/well adding the 10 μl of CCK-8 (Dojindo Molecular Technologies, Kumamoto, Japan). Cell viability was monitored by detecting absorbance at 450 nm.

### Colony formation assay

Cell samples were transplanted into six-well culture plates for 14 days of incubation at 37°C with 5% CO_2_. Followed by treating with Crystal Violet solution, colonies with >50 cells were counted.

### Flow cytometry analysis of cell apoptosis

After transfection and/or 5-FU treatment, cell samples were re-suspended in the Binding buffer which contained Annexin-V fluorescein isothiocyanate (FITC)/propidium iodide (PI) dual staining kit (BD Biosciences, San Jose, CA) for 15 min. Samples were subjected to flow cytometer apoptosis analysis by FACS cytometry (BD Biosciences).

### Transwell invasion assay

A total of 1 × 10^5^ cell samples of different groups were planted into the top chamber of Transwell insert (Corning Incorporated, Corning, NY) which was coated with Matrigel membrane (BD Biosciences). Lower chamber was filled with complete medium. Twenty-four hours later, invaded cell samples were cultured with Crystal Violet and exposed to microscope (magnification, ×200).

### Tube formation assay

Matrigel was prepared on ice, then transferred to 48-well plates for solidifying. Colon cancer cell samples diluted to 4 × 10^5^ cells/well in 48-well plates were cultivated at 37°C in 5% CO_2_ atmosphere for 24 h. Samples were finally observed through microscope.

### RNA immunoprecipitation assay

For Ago2-RNA immunoprecipitation (RIP), EZ-Magna RIP RNA Binding Protein Immunoprecipitation Kit (Millipore, Bedford, MA) was commercially acquired and utilized as instructed by supplier. The anti-Ago2 antibody and control anti-IgG antibody (Millipore) were employed. For MS2-RIP, pMS2-GFP and pSL-MS2, pSL-LINC01123 were co-transfected into cell samples as per manual. Precipitates were processed by quantitative real-time PCR (qRT-PCR).

### Dual-luciferase reporter assay

The wildtype (Wt) and mutated (Mut) miR-34c-5p binding sites to LINC01123 or VEGFA fragments were prepared for forming the reporter vectors LINC01123-Wt/Mut and VEGFA-Wt/Mut using pmirGLO vector (Promega, Madison, WI). The recombinant reporter vectors were co-transfected with indicated transfection plasmids in colon cancer cell samples for 48 h, then exposed to Dual-Luciferase Reporter Assay System (Promega).

### Subcellular fractionation

Colon cancer cell samples were collected for treating with cell fractionation buffer and cell disruption buffer in sequence. After isolating cytoplasmic and nuclear fractions, LINC01123 expression was quantified by qRT-PCR.

### RNA pull down assay

The miR-34c-5p fragments covering the putative mRNAs binding sites were acquired and biotin-tagged into miR-34c-5p biotin probe. Protein extracts from colon cancer cells were mixed with miR-34c-5p biotin probe or miR-34c-5p non-biotin probe, and beads for 1 h, qRT-PCR was followed.

### Western blot

Protein samples were extracted from RIPA lysis buffer, then treated with 12% SDS/PAGE and transferred electrophoretically to PVDF membranes. Primary antibodies against VEGFA, cleaved caspase 3, total caspase 3 and loading control GAPDH or Tubulin were procured from Abcam (Cambridge, MA, U.S.A.) and applied after treating membranes with 5% skim milk. Following washing in TBST, the HRP-tagged secondary antibody was utilized, the exposed to ECL chemiluminescent detection system (Thermo Fisher Scientific, Rochester, NY).

### Statistical analysis

Results of all independent bio-triplications were expressed as the mean ± standard deviation (SD) for each assay. Prism version 5.0 (GraphPad Software, La Jolla, CA) was used to process experimental data with Student’s *t* test or one-way ANOVA. *P*-value threshold was set as 0.05 to indicate statistical significance.

## Results

### The up-regulated LINC01123 facilitates cellular processes and chemoresistance in colon cancer

**Figure 1:**
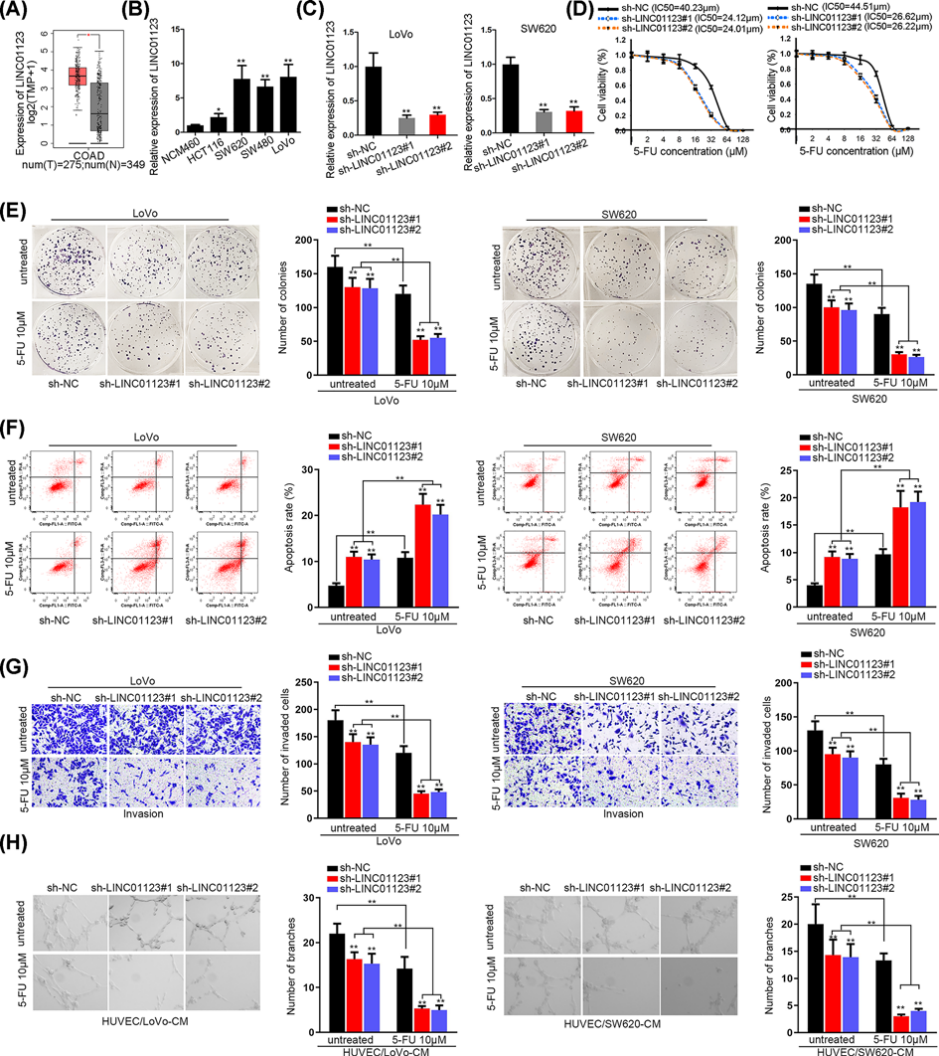
The up-regulated LINC01123 facilitates cellular process and chemoresistance in colon cancer (**A**) TCGA and GTEx databases of LINC01123 expression in COAD. (**B**) qRT-PCR of LINC01123 level in colon cancer cells and NCM460 cells. (**C**) qRT-PCR of the interference efficiency of LINC01123. (**D**) CCK-8 assay of cell proliferation in LoVo and SW620 cells with silenced LINC01123 and the addition of 5-FU. (**E**) Colony formation assay of the number of colonies in transfected cells treated with 10 μM 5-FU. (**F**) Flow cytometry assay of cell apoptosis in sh-LINC01123#1/2-transfected and 5-FU-treated cells. (**G**) Transwell assay of cell invasive ability in different groups. (**H**) Tube formation assay of angiogenesis in transfected HUVEC cells with the addition of 5-FU or not. *P<0.05, **P<0.01.

We first employed TCGA and GTEx databases to analyze the expression of LINC01123 in colon adenocarcinoma (COAD). The results revealed that LINC01123 was evidently up-regulated in COAD (Figure 1A). As for the existence of different transcripts of lncRNAs, we measured the expression level of two transcripts of LINC01123 in HCT116, SW620, SW480 and LoVo cells as well as in normal colon epithelial cell NCM460. As shown in Figure 1B, LINC01123-201 (named as LINC01123 in the whole manuscript) manifested conspicuous higher expression in colon cancer cells relative to NCM460 cells. However, another transcript LINC01123-202 presented no significant difference in normal NCM460 cell and CRC cells (Supplementary Figure S1A). Therefore, functional assays were carried out and LINC01123 was knocked down for preparation. qRT-PCR analyzed that the transfection of sh-LINC01123#1/2 induced a dramatic decrease in the expression of LINC01123 (Figure 1C). Then, CCK-8 assay demonstrated that LINC01123 silencing caused a reduction in IC_50_ value of cells to 5-FU treatment (Figure 1D). Considering the IC_50_ value of different cells, we chose 10 μM 5-FU for all subsequent experiments. In colony formation assay, it could be explicitly observed that LINC01123 depletion suppressed colongenic ability of control cells, and this suppressive effect was more obvious in cells treated with 10 μM of 5-FU (Figure 1E). Afterward, flow cytometry analysis indicated that cell apoptosis was enhanced by LINC01123 deficiency and further expedited by 5-FU treatment (Figure 1F). The protein level of cleaved-caspase 3 was increased in LINC01123-impeded cells and increased more significance in 5-FU-treated cells (Supplementary Figure S1B). Moreover, the number of invaded cells was decreased by the knockdown of LINC01123 and addition of 5-FU (Figure 1G). In the end, angiogenesis was also proved to be restrained in sh-LINC01123#1-transfetced cells and restrained more efficient in cells supplemented with 10 μM 5-FU (Figure 1H). These results revealed that LINC01123 depletion contributed to sensitizing CRC cells to 5-FU.

### MiR-34c-5p interacts with LINC01123 and exerts anti-tumor role in CRC

**Figure 2:**
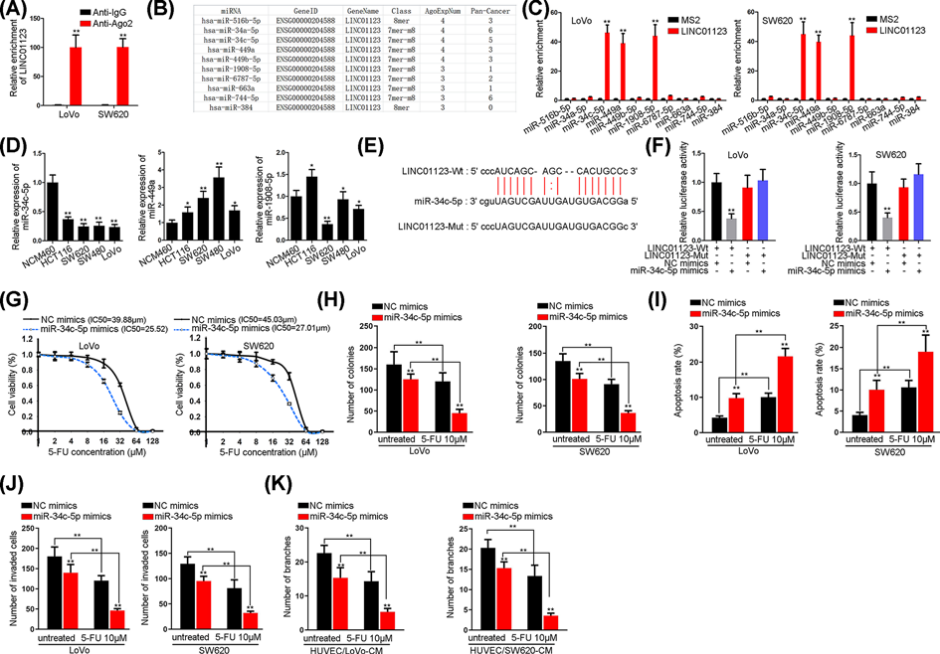
MiR-34c-5p is sponged by LINC01123 and promotes colon cancer cells progression as well as chemosensitivity (**A**) RIP assay of the relationship between Ago2 and LINC01123. (**B**) ENCORI of ten miRNAs which might bind with LINC01123. (**C**) MS2-RIP assay of the affinity between LINC01123 and ten candidate miRNAs. (**D**) qRT-PCR of the expression of three screened out miRNAs in colon cancer cells and NCM460 cells. (**E**) The binding sites between LINC01123 and miR-34c-5p. (**F**) Luciferase reporter assay of the binding between LINC01123 and miR-34c-5p. (**G**) CCK-8 assay of cell proliferative ability in cells with overexpressed miR-34c-5p and different concentrations of 5-FU. (**H**) Colony formation assay of the clonogenic ability in transfected cells with the treatment of 10 μM 5-FU. (**I**) Flow cytometry assay of cell apoptotic ability in different groups. (**J**) Transwell assay of cell invasion in miR-34c-5p mimics-transfected and 5-FU-treated cells. (**K**) Tube formation assay of angiogenesis in transfected HUVEC cells with or without the treatment of 5-FU. *P<0.05, **P<0.01.

In order to verify the hypothesized ceRNA role of LINC01123, Ago2-RIP assay was utilized to figure out the relationship between LINC01123 and Ago2, indicating its existence in RISC. As shown in Figure 2A, the binding between LINC01123 and Ago2 was validated. Therefore, we went on to search for the miRNAs which could bind with LINC01123. A total of ten miRNAs were screened out by ENCORI website (http://starbase.sysu.edu.cn/) with a condition of CLIP data: high stringency ≥ 3 (Figure 2B). In subsequence, MS2-RIP assay validated that miR-34c-5p, miR-449a and miR-1908-5p could bind with LINC01123 (Figure 2C). Afterward, qRT-PCR analysis indicated that miR-34c-5p was remarkably down-regulated in colon cancer cells, while the other two miRNAs presented no significant change (Figure 2D). Therefore, miR-34c-5p was chosen to be investigated and the binding sites between miR-34c-5p and LINC01123 were probed (Figure 2E). Then, the results in Figure 2F manifested that miR-34c-5p overexpression impaired the luciferase activity of LINC01123-Wt reporter. Furthermore, it was verified that miR-34c-5p overexpression could decrease the IC_50_ value of CRC cells to 5-FU (Figure 2G). Moreover, the number of colonies reduced by miR-34c-5p could be further decreased in cells treated with 5-FU (Figure 2H and Supplementary Figure S2A), suggesting that miR-34c-5p could suppress cell proliferation and reverse chemoresistance. In addition, cell apoptosis was unveiled to be motivated by miR-34c-5p overexpression and higher apoptosis rate was measured in cells treated with 10 μM 5-FU (Figure 2I and Supplementary Figure S2B). More importantly, enforced expression of miR-34c-5p in combination with 10 μM 5-FU could substantially inhibit cell invasive ability (Figure 2J and Supplementary Figure S2C). Finally, tube length was also disclosed to be reduced by miR-34c-5p overexpression and 10 μM 5-FU addition (Figure 2K and Supplementary Figure S2D).

### VEGFA is regulated by miR-34c-5p and positively correlated with LINC01123 expression

**Figure 3:**
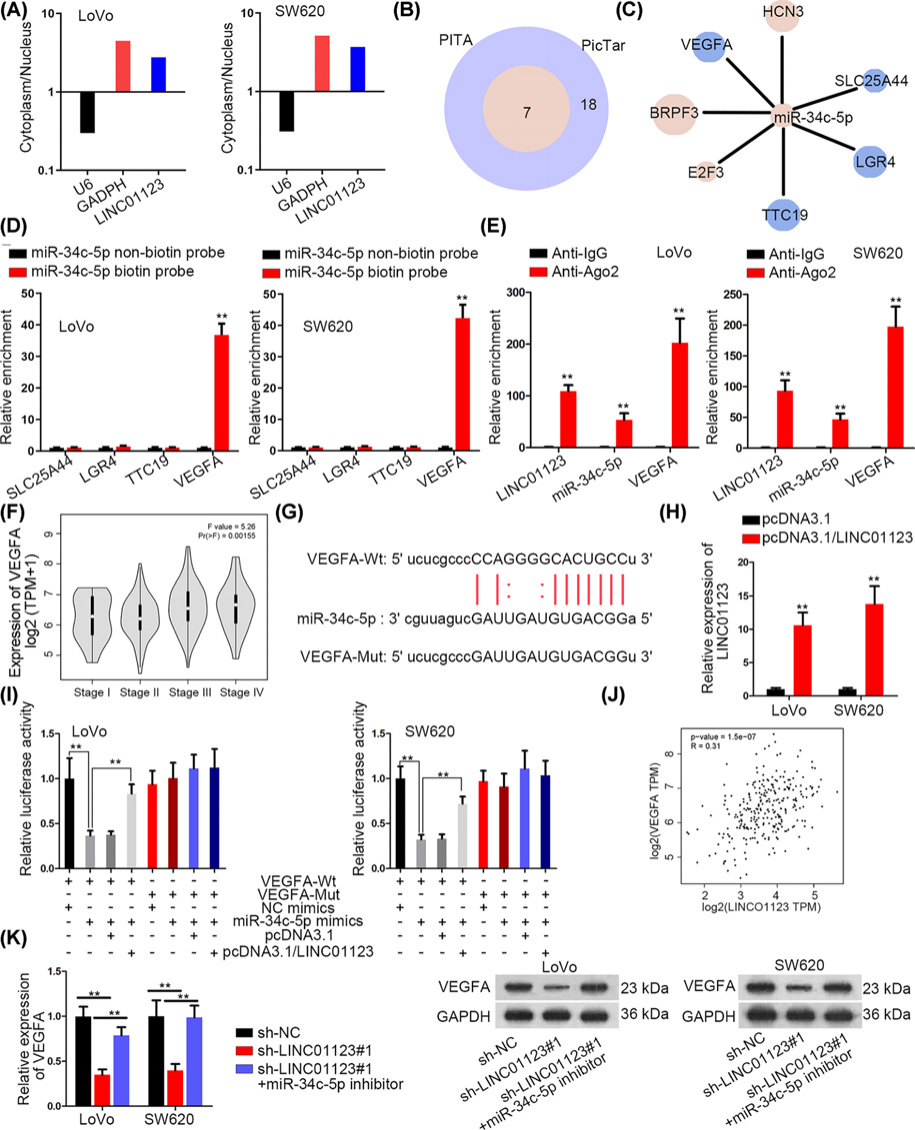
VEGFA is regulated by miR-34c-5p and positively correlated with LINC01123 expression (**A**) Subcellular fractionation assay of the localization of LINC01123. (**B**) Venn diagram of the mRNAs that may bind with miR-34c-5p. (**C**) qRT-PCR of seven candidate mRNAs expression in cells transfected with miR-34c-5p mimics. Red represents up-regulation while blue represents down-regulation. The size of the circle represents fold-change. (**D**) RNA pull down assay of the combination between miR-34c-5p and four candidates. (**E**) RIP assay of the interaction between miR-34c-5p and VEGFA. (**F**) GEPIA website of the correlation between VEGFA expression and different COAD stages. (**G**) The binding sites between miR-34c-5p and VEGFA. (**H**) qRT-PCR of overexpression efficiency of LINC01123. (**I**) Luciferase reporter assay of the combination between miR-34c-5p and VEGFA. (**J**) TCGA database of the correlation between VEGFA and LINC01123 expression. (**K**) qRT-PCR and Western blot assays of VEGFA expression in different transfected cells. **P<0.01.

Subcellular fractionation assay was performed to study whether LINC01123 was qualified to engage in post-transcriptional regulation. The results revealed that LINC01123 was mainly located in the cytoplasm of LoVo and SW620 cells (Figure 3A). Subsequently, ENCORI was utilized to figure out the mRNAs which could be regulated by miR-34c-5p. As illustrated in Figure 3B, by intersecting the data obtaining from PITA and PicTar databases, seven mRNAs were spotted. And four of them were analyzed to be down-regulated when miR-34c-5p was overexpressed (Figure 3C). Furthermore, only the predicted affinity between VEGFA and miR-34c-5p was verified via RNA pull down assay (Figure 3D). Besides, RIP assay confirmed the coexistence of LINC01123, miR-34c-5p and VEGFA in RISC (Figure 3E). Then, it was searched in GEPIA that VEGFA expression was correlated with different tumor stages of COAD (Figure 3F). The binding sites between miR-34c-5p and VEGFA were obtained (Figure 3G). Afterward, LINC01123 was successfully overexpressed in two colon cancer cells (Figure 3H). Next, the impaired luciferase activity of VEGFA-Wt reporter triggered by miR-34c-5p overexpression was reversed by enforced expression of LINC01123 (Figure 3I). More importantly, the positive expression correlation between VEGFA and LINC01123 was analyzed based on the COAD data of GEPIA (Figure 3J). The regulatory role of LINC01123 in VEGFA expression was further revealed and the result showed that the mRNA and protein levels of VEGFA were restrained in sh-LINC01123#1-transfetced cells and this restraint could be countervailed by miR-34c-5p inhibition (Figure 3K).

### LINC01123/miR-34c-5p/VEGFA axis enhances colon cancer development and chemoresistance

**Figure 4:**
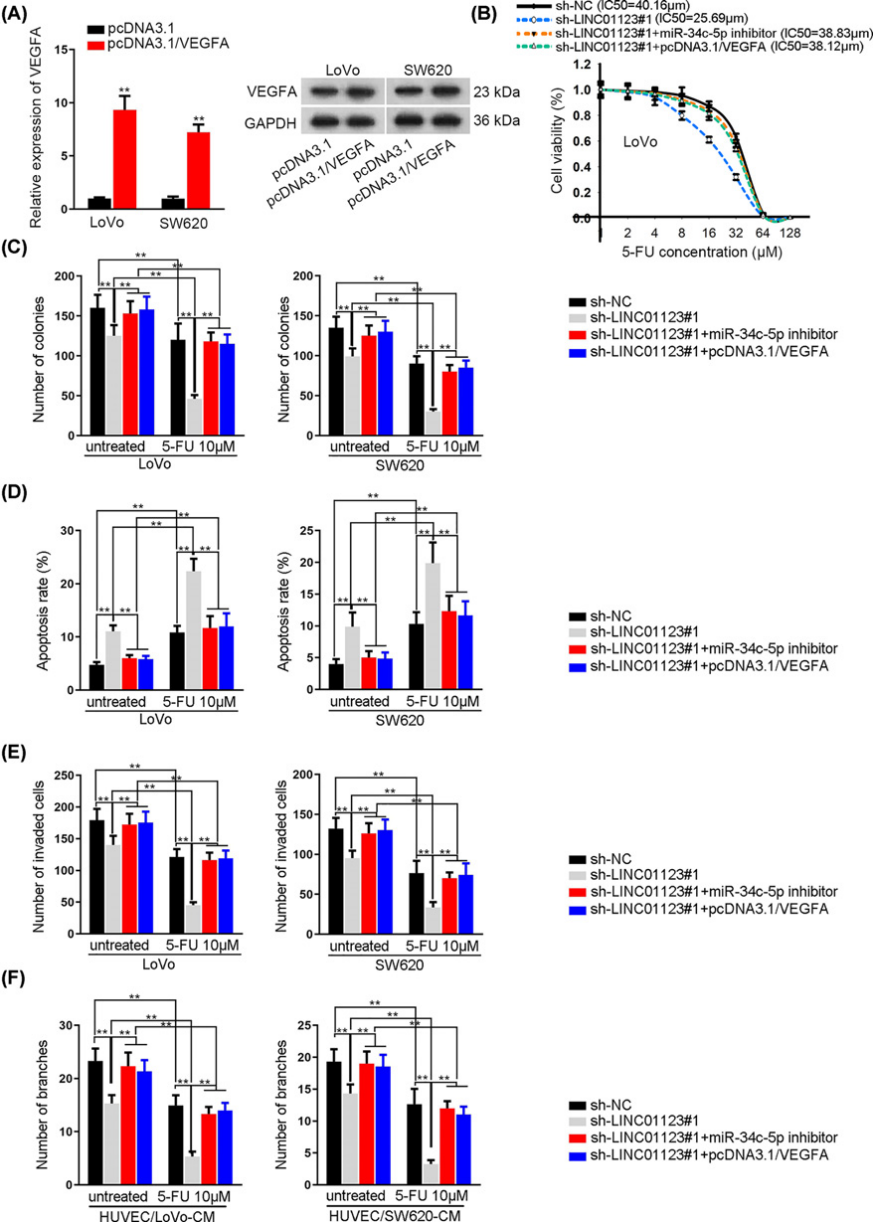
LINC01123/miR-34c-5p/VEGFA axis enhances colon cancer development and chemoresistance (**A**) qRT-PCR and Western blot assays of the overexpression efficiency of VEGFA. (**B**) CCK-8 assay of cell proliferation in different groups. (**C**) Colony formation assay of cell proliferative ability in different transfected cells with or without 10 μM 5-FU. (**D**) Flow cytometry assay of cell apoptosis in different groups. (**E**) Transwell assay of the number of invaded cells in different groups. (**F**) Tube formation assay of angiogenesis in transfected HUVEC cells added with 5-FU or not. **P<0.01.

The function of LINC01123/miR-34c-5p/VEGFA axis was further analyzed by rescue assays. Before functional assays, VEGFA was overexpressed in indicated CRC cells (Figure 4A). The IC_50_ value of LoVo and SW620 cells to 5-FU decreased by LINC01123 was further recovered by the inhibition of miR-34c-5p and overexpression of VEGFA (Figure 4B). Then, colony formation assays disclosed that cell proliferation was hindered by LINC01123 deficiency, while this obstruction could be mostly reversed by miR-34c-5p inhibition or VEGFA overexpression (Figure 4C and Supplementary Figure S3A). Moreover, the facilitated cell apoptosis in sh-LINC01123#1-transfetced cells could be restored by miR-34c-5p silencing or VEGFA overexpression, treated with 5-FU or not (Figure 4D and Supplementary Figure S3B). In addition, the impaired cell invasive ability triggered by LINC01123 shortage could be compensated by miR-34c-5p knock down or VEGFA overexpression, and these effects were more obvious in 5-FU-treated group (Figure 4E and Supplementary Figure S3C). Finally, it was revealed that shortage of miR-34c-5p or enforced expression of VEGFA could rescue the LINC01123 depletion-mediated angiogenesis, especially in cells treated with 5-FU (Figure 4F and Supplementary Figure S3D).

## Discussion

Among the chemotherapeutic agents targeted at colon cancer, 5-FU is the most widely utilized [16]. When colon cancer patients at advanced stages were treated with 5-FU, tumor size was detected to be reduced by approximately half and their median survival was analyzed to be prolonged by 5 months [17]. Nevertheless, 5-FU treatment was revealed to induce therapy resistance [18,19] accompanied by several side effects [16,20]. Therefore, it is of consequence to reveal the molecular mechanism underlying cell resistance to 5-FU as well. Similar to the up-regulated expression in COAD, LINC01123 was also highly expressed in colon cancer cells. More importantly, LINC01123 silencing was disclosed to pose suppressive influences on cell proliferation, invasion and angiogenesis. Moreover, this suppression was more evident when the transfected cells were treated with 10 μM 5-FU. In conclusion, LINC01123 promotes the malignancy of colon cancer and enhances the resistance of colon cancer cells to 5-FU.

Subsequently, it was disclosed that LINC01123 existed in RISC, indicating that LINC01123 could bind with certain miRNAs. The regulation of lncRNA on miRNA has been proposed and confirmed. Through the miRNA binding elements, lncRNA could compete with mRNA to combine with miRNA. In this way, mRNA is also freed from the regulation of upstream miRNA. In our work, miR-34c-5p was identified as the miRNA that could bind with LINC01123. MiR-34c-5p could down-regulate AREG-EGFR-ERK pathway to suppress ovarian cancer stemness [21]. In colorectal cancer, miR-34c-5p promoter was methylated, therefore miR-34c-5p was down-regulated [22]. Besides, Pien Tze Huang was suggested to up-regulate miR-34c-5p, thereby restraining colorectal cancer cell proliferation [23]. This work illustrated that miR-34c-5p expression was lower in colon cancer cells. In addition, the combination between miR-34c-5p and LINC01123 was verified. Furthermore, miR-34c-5p could suppress colon cancer cells cellular process and chemoresistance. In subsequence, LINC01123 was observed to be occupied more in the cytoplasm. And VEGFA was uncovered as mRNA which competed with LINC01123 to bind with miR-34c-5p. VEGFA was reported to promote angiogenesis in cancers, including colon cancer [24,25]. Evidences in this research manifested that VEGFA was correlated with different stages in COAD. Besides, the binding between VEGFA and miR-34c-5p was corroborated. And VEGFA was supported to be positively regulated by LINC01123 at both mRNA and protein levels. In the end, rescue assays illustrated that LINC01123 regulates the development and chemoresistance of colon cancer cells dependent on miR-34c-5p and VEGFA.

In conclusion, this work uncovered the role of LINC01123 in colon cancer, providing a new perspective into colon cancer treatment. LINC01123 exerted as a miR-34c-5p sponge in colon cancer to promote VEGFA-triggered colon cancer malignancy and chemoresistance.

## Supplementary Material

Supplementary Figures S1-S3

Supplementary Tables S1-S2

## Abbreviations

Ago2: argonaute 2
AREG-EGFR-ERK: amphiregulin-epidermal growth factor receptor-extracellular signal-regulated kinase
CCK-8: cell counting kit-8
ceRNA: competing endogenous RNA
COAD: colon adenocarcinoma
CRC: colorectal cancer
EZH2: enhancer of zeste 2 polycomb repressive complex 2 subunit
GAPDH: glyceraldehyde-3-phosphate dehydrogenase
GEPIA: Gene Expression Profiling Interactive Analysis
GTEx: Genotype-Tissue Expression
HRP: horse radish peroxidase
HUVEC: Human Umbilical Vein Endothelial Cells
LINC01123: long intergenic non-protein coding RNA 1123
lncRNA: long non-coding RNA
miRNA: microRNA
mRNA: messenger RNA
mut: mutant
NC: negative control
Pen/Strep: penicillin/streptomycin
qPCR: quantitative polymerase chain reaction
qRT-PCR: quantitative real-time PCR
RIP: RNA immunoprecipitation
RISC: RNA-induced silencing complex
TBST: Tris Buffered saline Tween
TCGA: The Cancer Genome Atlas
VEGFA: vascular endothelial growth factor A
Wt: wild type
5-FU: 5-Fluorouracil
