# Supplementary material for: LINC01123 facilitates proliferation, invasion and chemoresistance of colon cancer cells
Source: Biosci Rep. 2020 Aug 7;40(8):BSR20194062. doi: 10.1042/BSR20194062 (PMC7414518; doi:10.1042/BSR20194062)
Supplement: Supplementary Figures S1-S3 [file BSR-2019-4062_supp.pdf]

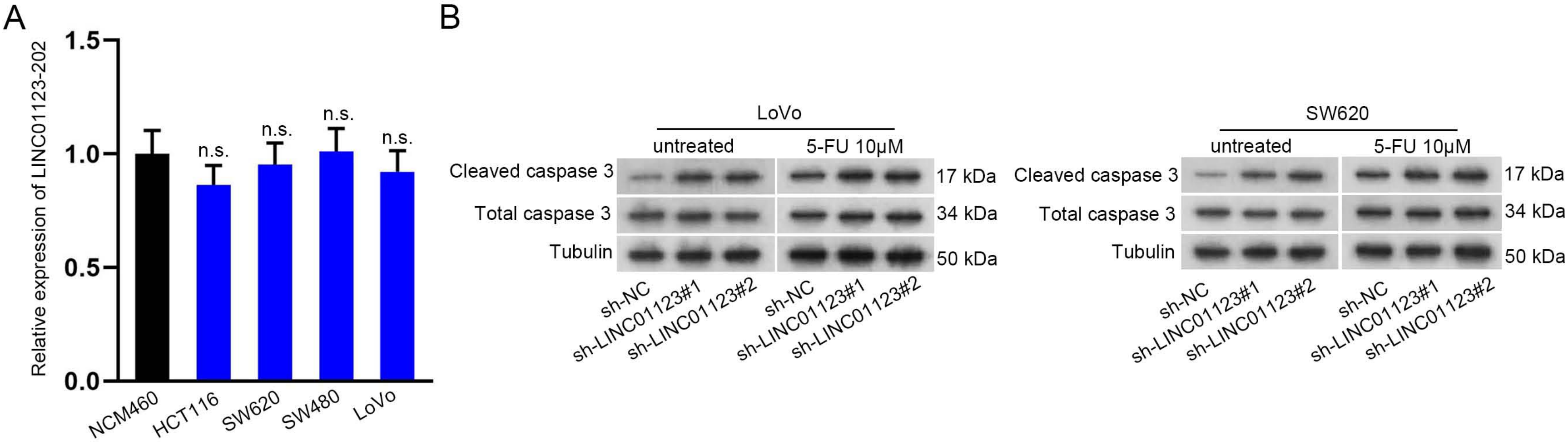
A
B
Relative expression of LINC01123-202
1.5
1.0
0.5
0.0
n.s.
n.s.
n.s.
n.s.
NCM460
HCT116
SW620
SW480
LoVo
LoVo
SW620
untreated
5-FU 10μM
Cleaved caspase 3
Total caspase 3
Tubulin
17 kDa
34 kDa
50 kDa
sh-NC
sh-LINC01123#1
sh-LINC01123#2

Figure S1: (A) qRT-PCR analysis of LINC01123-202 expression in four colon cancer cells and normal cell line NCM460. (B) The protein level of cleaved or total caspase-3 in LoVo and SW620 cells treated with or 5-FU after transfected with sh-NC or LINC01123-specific shRNAs. n.s. indicated data had no statistical significance.

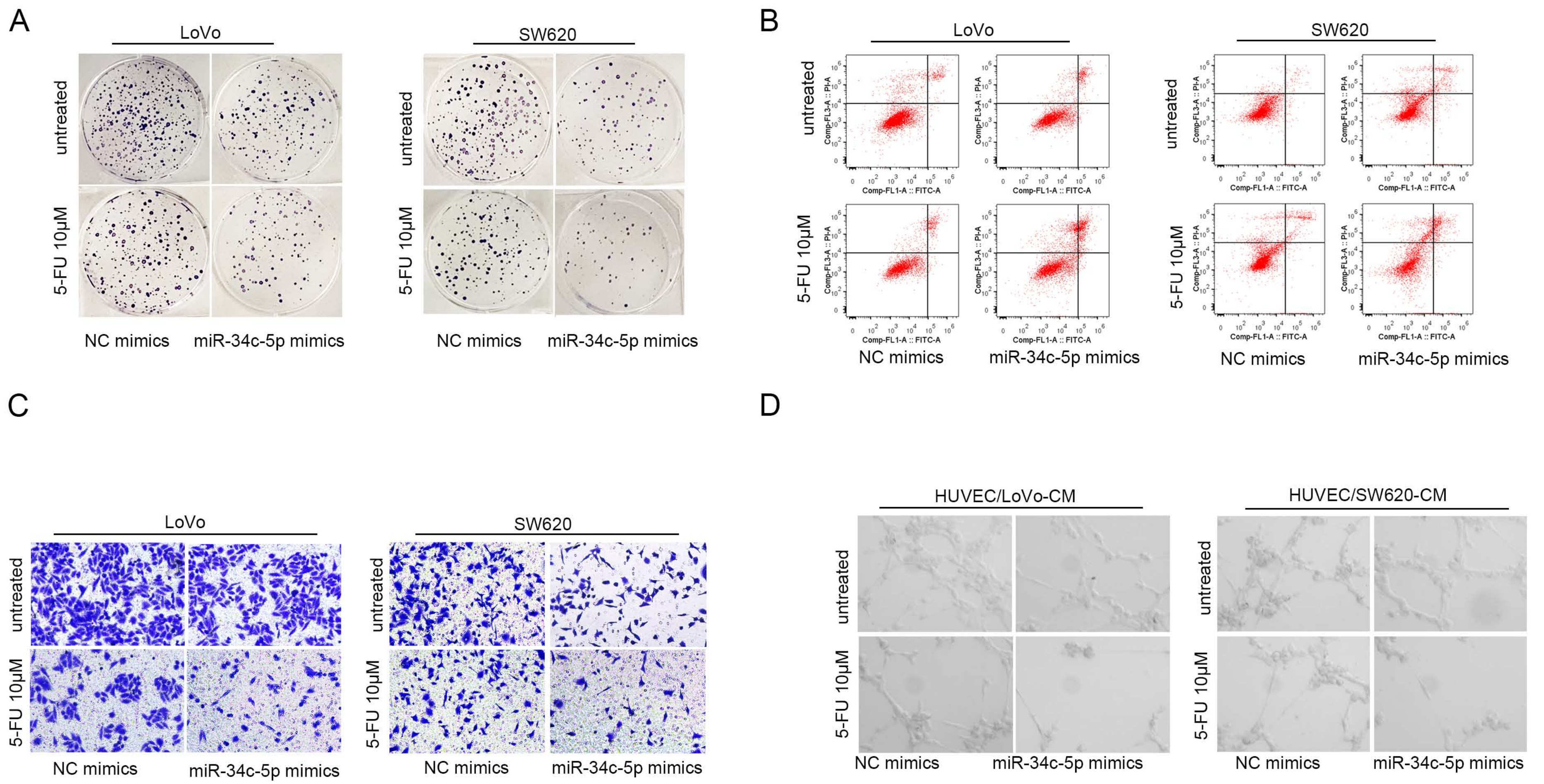

A
LoVo
SW620
untreated
5-FU 10μM
NC mimics
miR-34c-5p mimics
B
LoVo
SW620
Comp-FL3-A :: PI-A
Comp-FL1-A :: FITC-A
C
D
HUVEC/LoVo-CM
HUVEC/SW620-CM

Figure S2: (A-D)Experimental images of Figure 2H, I, J, K.

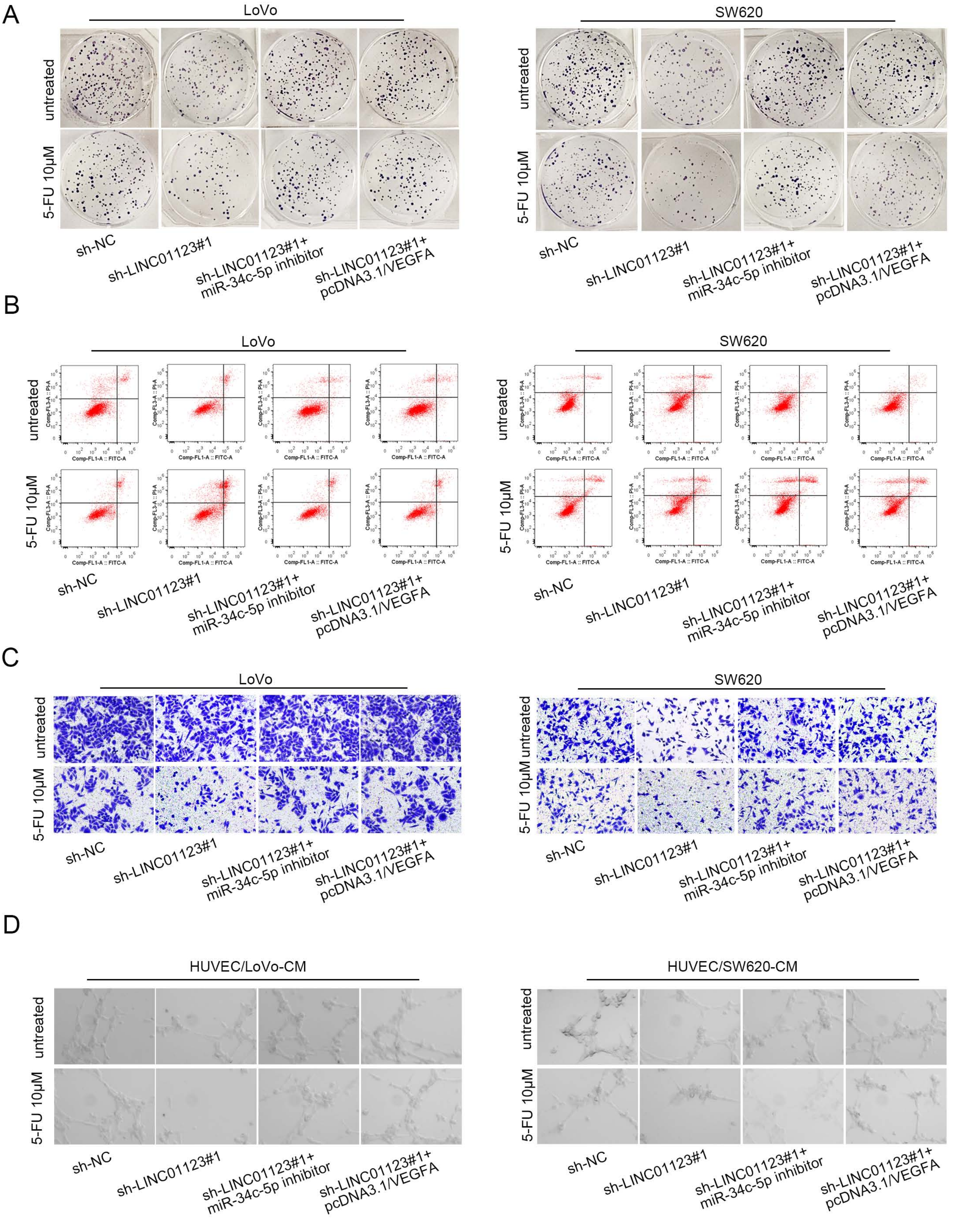
A
LoVo
SW620
untreated
5-FU 10μM
sh-NC
sh-LINC01123#1
sh-LINC01123#1+ miR-34c-5p inhibitor
sh-LINC01123#1+ pcDNA3.1/VEGFA
B
LoVo
SW620
untreated
5-FU 10μM
Comp-FL1-A :: FITC-A
Comp-FL3-A :: PI-A
sh-NC
sh-LINC01123#1
sh-LINC01123#1+ miR-34c-5p inhibitor
sh-LINC01123#1+ pcDNA3.1/VEGFA
C
LoVo
SW620
untreated
5-FU 10μM
sh-NC
sh-LINC01123#1
sh-LINC01123#1+ miR-34c-5p inhibitor
sh-LINC01123#1+ pcDNA3.1/VEGFA
D
HUVEC/LoVo-CM
HUVEC/SW620-CM
untreated
5-FU 10μM
sh-NC
sh-LINC01123#1
sh-LINC01123#1+ miR-34c-5p inhibitor
sh-LINC01123#1+ pcDNA3.1/VEGFA

Figure S3: (A-D)Experimental images of Figure 4C, D, E, F.
